# MgAl and ZnAl-Hydrotalcites as Materials for Cosmetic and Pharmaceutical Formulations: Study of Their Cytotoxicity on Different Cell Lines

**DOI:** 10.3390/ph15070784

**Published:** 2022-06-23

**Authors:** Maria Rachele Ceccarini, Matteo Puccetti, Cinzia Pagano, Morena Nocchetti, Tommaso Beccari, Alessandro di Michele, Maurizio Ricci, Luana Perioli

**Affiliations:** 1 Department of Pharmaceutical Sciences, University of Perugia, 06123 Perugia, Italy; mariarachele.ceccarini@unipg.it (M.R.C.); matteo.puccetti@gmail.com (M.P.); morena.nocchetti@unipg.it (M.N.); tommaso.beccari@unipg.it (T.B.); maurizio.ricci@unipg.it (M.R.); luana.perioli@unipg.it (L.P.); 2 Department of Physics and Geology, University of Perugia, 06123 Perugia, Italy; alessandro.dimichele@unipg.it

**Keywords:** hydrotalcites, layered double hydroxides, MgAl-HTlc, ZnAl-HTlc, HaCat, fibroblasts, HepG2, cytotoxicity

## Abstract

The knowledge about the effect of hydrotalcites (HTlcs), largely used in pharmaceutics, on non-malignant cell lines is limited. The effect of MgAl-HTlc-and ZnAl-HTlc- (NO^3−^/Cl^−^/CO_3_^2−^) on the cell viability of HaCat, fibroblasts and HepG2 was studied by MTT assay. Cells were incubated either with HTlc suspensions in the culture media and with the supernatant obtained from the suspension being centrifuged. MgAl-HTlcs suspensions resulted in being cytotoxic. As SEM and TEM analyses showed the presence of sub-micrometric particles in all the MgAl-HTlc examined, it could be hypothesized that this fraction can be internalized into cells reducing the viability. MgAl-HTlc-NO_3_ is the most cytotoxic probably due to the additional effect of NO_3_^−^ anions. ZnAl-HTlcs are cytotoxic, especially for HaCat and HepG2 cells (viability <60% at all the concentrations assayed). The effect is attributable both to the sub-micrometric fraction (identified by TEM) and to the high Zn^2+^ levels found in the culture medium by ICP-OES analysis, suggesting that ZnAl-HTlcs are less stable than MgAl-HTlc in the used media. The obtained results suggest that it is very important to perform ad hoc studies in order to evaluate HTlc safety before to be introduced in a formulation.

## 1. Introduction

Hydrotalcites (HTlc) are anionic clays, showing a typical lamellar structure, able to host exchangeable inorganic or organic anions. The lamellae are constituted by metal cations occupying the centers of octahedra, whose vertices contain hydroxide ions. Each OH group is shared by three octahedral cations and the hydrogen points towards the interlayer region similar to brucite, Mg(OH)_2_. The general formula of synthetic hydrotalcite-like compound is [M(II)_1−x_M(III)_x_(OH)_2_]^x+^(An_−x/n_)^x−^ mS, where M(II) is a divalent metal cation (usually Mg^2+^, Zn^2+^) and M(III) is a trivalent metal cation (usually Al^3+^, Fe^3+^). Generally, the x value, M(III)/M(II) + M(III) ranges between 0.25 and 0.40, A^n−^ is an exchangeable inorganic or organic anion that compensates for the positive charge generated from the aluminum of the layer, m values express the mol of solvent, S, usually, water, co-intercalated per mole of compound [1].

The layered structure, together with the ability to host anionic molecules, makes HTlc versatile materials that can find applications in many fields [2]. Among the many uses of HTlcs in pharmaceutics are those related to their being: (i) starting materials for the intercalation of active pharmaceutical ingredients (drug delivery systems) [3,4], (ii) active ingredients as antacids due to the high buffering capacity [5] and adsorbents nature [6], and (iii) rheological agents and fillers [7,8,9].

At present, HTlcs can be found in products commercially available as: (i) antiacid (MgAl-HTlc-CO_3_) to relieve heartburn and acid-related stomach complaints; (ii) antiperspirants and adsorbents (in deodorants-ZnAl-HTlc-CO_3_); (iii) as matting and rheological agents as well as a physical UV filter in skincare and makeup products.

Due to such many applications involving human health, it might be necessary to investigate in depth the profile of HTlc safety. Limited data available in the literature suggest that it is not possible to classify peremptorily HTlc as either “safe” or “toxic“ in that cytotoxicity assay results are influenced by particle size, chemical composition and chemical stability [10,11,12]. In addition, the treatment of cell lines coming from various sources often produces different responses. Nonetheless, HTlcs have been extensively studied as delivery system in cancer therapy, because of their ability to penetrate into tumor cells and to provoke their death [13,14,15]. On the other hand, there are several conditions—as mentioned above—where cytotoxicity to human cells would not be a desirable effect, and rather compounds should be selected based on the lack of cytotoxic effects.

The aim of this work was to investigate the in vitro effect of two HTlc types (MgAl-HTlc and ZnAl-HTlc) on the viability of three different cell lines. These lamellar materials, intercalating the inorganic anions NO_3_^−^, Cl^−^ or CO_3_^2−^, are largely used in pharmaceutical, cosmetic, and nutraceutical fields. As HTlcs are largely used in formulations for skin and oral use, the cell lines used for these studies were keratinocytes (HaCat) as representative for epidermis, fibroblasts as representative for derma and, hepatocytes (HepG2), representative for the liver tissue. Liver is indeed the first organ accessed by xenobiotics through the GI tract. The study aimed to evaluate the effect on the cell viability on the mentioned cell lines, considering the influence of their chemical composition, particle size, and chemical stability.

## 2. Results and Discussions

After synthesis, the different HTlcs, Zn/MgAl-HTlc in carbonate, chloride and nitrate forms, were submitted for a preliminary characterization. The X-Ray Powder Diffraction (XRPD) profiles allowed us to recognize the typical pattern of the HTlc structure and, from the interlayer distance that is correlated with the position of the first reflection, to get information on the anion located in the interlayer region. The interlayer distances of HTlc in carbonate, chloride, and nitrate form, reported in Table 1, agree with literature data [14]. By combining the results coming from ICP-OES and thermogravimetric analyses, we established the empirical formulas for all the synthesized HTlcs (Table 1).

The dimensional analysis (Table 2) showed that ZnAl-HTlc-CO_3_ particles are larger (13.82 µm) than the other HTlcs types, showing a value below 10 µm (Table 2).

**Figure 1:**
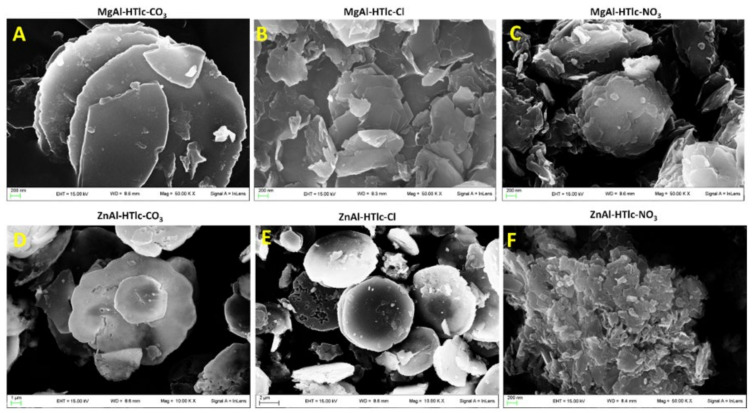
Micrographs obtained by SEM analysis of: MgAl-HTlc-CO_3_ (**A**); MgAl-HTlc-Cl (**B**); MgAl-HTlc-NO_3_ (**C**); ZnAl-HTlc-CO_3_ (**D**); ZnAl-HTlc-Cl (**E**); ZnAl-HTlc-NO_3_ (**F**).

**Figure 2:**
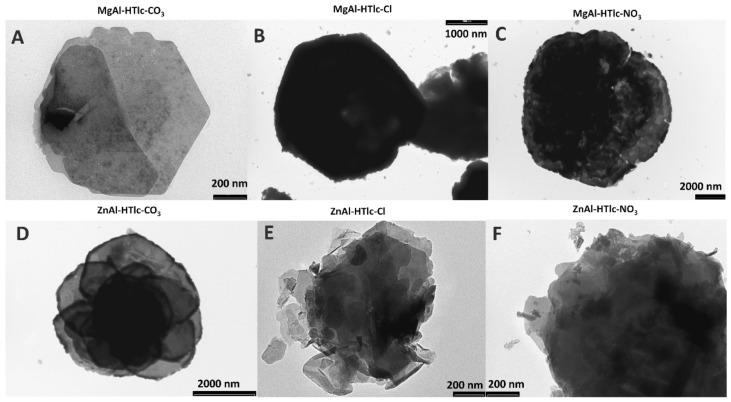
TEM images of: MgAl-HTlc-CO_3_ (**A**); MgAl-HTlc-Cl (**B**); MgAl-HTlc-NO_3_ (**C**); ZnAl-HTlc-CO_3_ (**D**); ZnAl-HTlc-Cl (**E**); ZnAl-HTlc-NO_3_ (**F**).

Morphological analysis, performed by both scanning electron microscopy (SEM, Figure 1) and transmission electron microscopy (TEM, Figure 2), allowed us to verify the main differences among the examined HTlc samples.

MgAl-HTlc micrographs (Figure 1A–C) show aggregates of particles having a platelet-like structure [16] with a diameter of about 3, 1, and 1.5 µm for CO_3_^2−^ (Figure 1A and Figure 2A), Cl^−^ (Figure 1B and Figure 2B) and NO_3_^−^ (Figure 1C and Figure 2C) respectively. In addition to micrometric crystals, in all the samples, nanometric fragments are well visible in all the samples. Moreover, TEM image shows jagged edges, and, again, the presence of small fragments is clearly detectable around the crystal particle (Figure 2).

Similar morphology was observed on SEM and TEM analyses, for both ZnAlHTlc-CO_3_ (Figure 1D and Figure 2D) and ZnAlHTlc-Cl (Figure 1E and Figure 2E) [17] although they show a larger average size, of 10 and 7 µm respectively. Conversely, SEM images of ZnAlHTlc-NO_3_ (Figure 1F) showed that the sample is constituted by flat and irregular crystals with an average dimension of about 500 nm [18]. Again, both SEM and TEM images showed the presence of nanometric fragments around the particle (Figure 1F and Figure 2F). The fraction of nanometric fragments seemed to increase, according to the sequence, ZnAlHTlc-CO_3_, ZnAlHTlc-Cl, ZnAlHTlc-NO_3_.

As the procedures followed for HTlcs synthesis are intended for the preparation of micrometric particles, it is clear that the submicrometric fraction is a by-product deriving from the synthetic preparation process or from the grinding procedures.

### 2.1. Cell Lines Choice

HTlcs are vastly employed in biologic and manufacturing settings as an active ingredient, excipient (filler, rheological agent), and drug carrier. For this reason, we evaluated their effect in vitro on the viability of three different cell lines: keratinocytes (HaCat), human fibroblasts, and hepatocytes (HepG2). Keratinocytes represent the first cells exposed to formulations applied to skin. Due to the large use of HTlcs in semisolid formulations both as a delivery system and as excipients (rheological, texturizing, or opacizing agents), keratinocytes are a subject of interest. Fibroblasts are the most representative cell type of the derma. Many products applied to the epidermis can cross this layer especially when it is compromised (e.g., by wounds or irritation), reaching the surrounding layer in which fibroblasts are localized. HTlcs have been also largely studied for products intended for oral administration. For example, MgAl-HTlc-CO_3_ is used in commercially available products as an antacid. The liver is the first organ encountered by xenobiotics after oral ingestion.

### 2.2. ICP-OES Analysis and pH Measurement

The stability of the selected HTlcs in cultured cell lines was firstly studied. DMEM was used as a culture medium for HaCat and fibroblasts and MEM for HepG2 showing both a pH value of 7.4. HTlcs were incubated for 24 h at 37°C with the culture media then the solid particles were separated by centrifugation and the supernatants were subjected both to pH measurement and to quantification of Mg^2+^, Zn^2+^ and Al^3+^ ions released from HTlcs. It is noteworthy that HTlcs only dissolve at pH < 5 and pH > 10 [19] suggesting that HTlcs should be stable in both DMEM and MEM. Instead, the pH measurements of the culture media incubated with HTlcs revealed that both the carbonate forms, MgAl-HTlc-CO_3_ and ZnAl-HTlc-CO_3_, produced an increase in pH values (Table 3) as a result of an occurring alkalinisation event.

This could be mainly attributed to the exchange of CO_3_^2−^ anions, located on the surface and on the edges of HTlc crystals, by the anions present in the DMEM and MEM. In all the other cases, no considerable changes in pH values were detected (Table 3). Metal ions quantification was performed using the culture media MEM and DMEM as a blank condition. Both media showed a very limited amount of Al^3+^ (~0.013 mg/L and 0.57 mg/L for MEM and DMEM respectively) and Mg (~23 mg/L for both media) while Zn^2+^ was absent.

**Figure 3:**
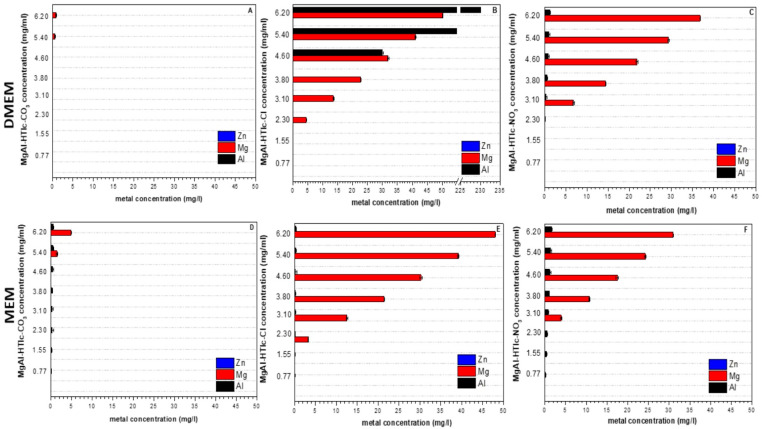
Concentrations of Al^3+^, Mg^2+,^ and Zn^2+^ more exceeding that found in the controls: MEM and DMEM. Supernatants of: (**A**) MgAl-HTlc-CO_3_ in DMEM; (**B**) MgAl-HTlc-Cl in DMEM; (**C**) MgAl-HTlc-NO_3_ in DMEM; (**D**) MgAl-HTlc-CO_3_ in MEM; (**E**) MgAl-HTlc-Cl in MEM; (**F**) MgAl-HTlc-NO_3_ in MEM.

From ICP OES analysis, MgAl-HTlc-CO_3_ resulted in the most stability in both MEM and DMEM. Figure 3 shows the concentration of metals exceeding the baseline (values of the blanks: MEM and DMEM). MgAl-HTlc-CO_3_ shows a small increase in Mg^2+^ levels only at the two highest concentrations (Figure 3A,D, 6.20 and 5.40 mg/mL). MgAl-HTlc-Cl was the most unstable showing important increases in Mg^2+^ levels in the concentration range of 6.20–2.30 mg/mL compared to the blanks (Figure 3B,E). As to MgAl-HTlc-NO_3_ Mg^2+^ concentrations were likewise high, in both DMEM and MEM, in the concentration range of 6.20–3.10 mg/mL (Figure 3C,F). These data suggest that during the incubation time (24 h) MgAl-HTlc-Cl and MgAl-HTlc-NO_3_ undergo partial solubilisation. In addition, in the case of Al^3+^, an increased concentration was observed, especially in the case of MgAl-HTlc-Cl samples prepared in DMEM (Figure 3B).

**Figure 4:**
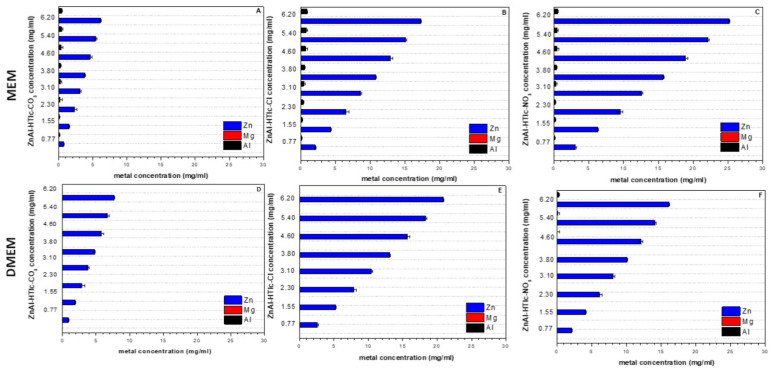
Concentrations of Al^3+^, Mg^2+,^ and Zn^2+^ more exceeding that found in the controls: MEM and DMEM. Supernatants of: (**A**) ZnAl-HTlc-CO_3_ in MEM; (**B**) ZnAl-HTlc-Cl in MEM; (**C**) ZnAl-HTlc-NO_3_ in MEM; (**D**) ZnAl-HTlc-CO_3_ in DMEM; (**E**) ZnAl-HTlc-Cl in DMEM; (**F**) ZnAl-HTlc-NO_3_ in DMEM.

In the case of ZnAl-HTlcs, Zn^2+^ (usually absent in the culture media) was found in all the samples being analysed (Figure 4). The carbonate form, produced the lowest levels both in MEM and in DMEM (Figure 4A,D respectively) due to the limited solubility. Zn concentration resulted particularly high for the supernatants obtained by suspending ZnAl-HTlc-NO_3_ in MEM (~25 mg/L, Figure 4C) and ZnAl-HTlc-Cl in DMEM (~21 mg/L) (Figure 4E). All concentrations, measured for both ZnAl-HTlc-Cl and ZnAl-HTlc-NO_3_, were particularly high in MEM (Figure 4B,C respectively).

### 2.3. Cytotoxicity Studies on MgAl-HTlc Samples

**Figure 5:**
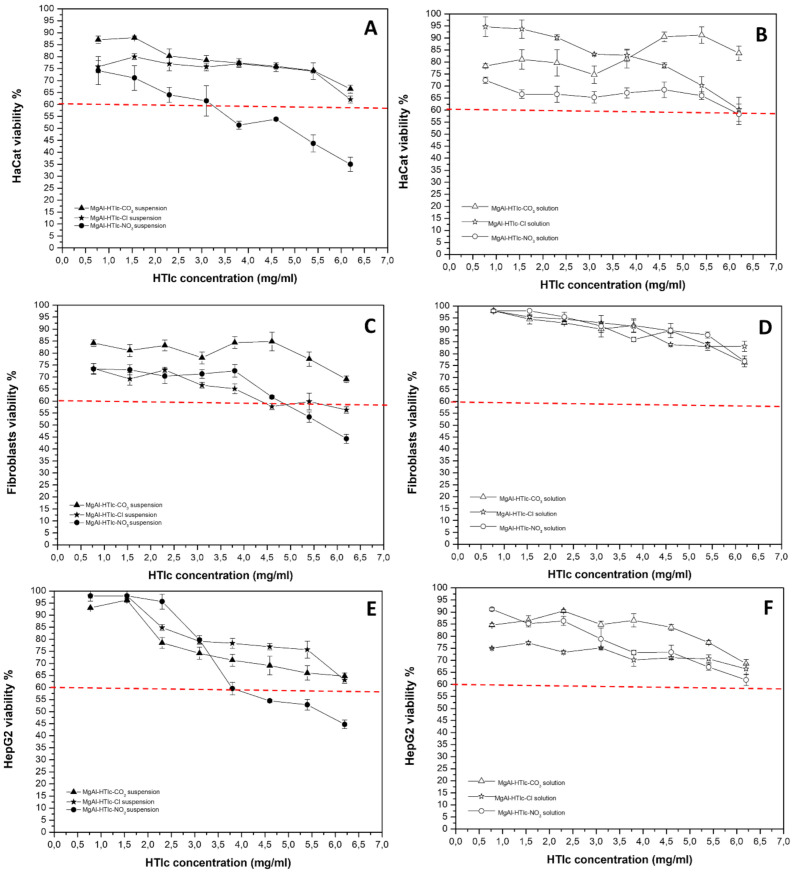
Viability of HaCat cells incubated with MgAl-HTlc suspensions (**A**) and supernatants (**B**); viability of fibroblasts incubated with MgAl-HTlc suspensions (**C**) and supernatants (**D**); viability of HepG2 cells incubated with MgAl-HTlc suspensions (**E**) and supernatants (**F**). The percentage of viable cells with respect to the control was reported as the mean ± SD of five independent experiments. Dotted lines indicate 60% cell viability.

HTlc samples, both suspensions and supernatants, were prepared according to the method described in par. 3.8, were assayed in terms of cytotoxicity on HaCat, fibroblasts, and HepG2 cells. Cell responses changed according to HTlc chemical composition, particle size, and chemical stability in the culture media, as well as to the different cell lines. Both MgAl-HTlc-CO_3_ and MgAl-HTlc-Cl suspensions were safe for HaCat cells at all the concentrations assayed, with cell viability > 65% (Figure 5A). In the case of MgAl-HTlc-NO_3_, two different trends were observed depending on the concentration range, safe between 0.77 and 3.10 mg/mL with cell viability > 60%, which decreased to 51–35% in the concentration range of 3.80–6.20 mg/mL (Figure 5A). This effect could be attributed to the suspended particles and/or to the solubilized metals. In order to better understand this, the cytotoxicity was evaluated for the supernatants separated from the different dilutions of the suspensions. The results (Figure 5B) showed that the MgAl-HTlc-NO_3_ supernatants were not harmful to HaCat cells, implying that the solid fraction is the main cause of cell death when incubated with the suspension in a concentration dependent manner (Figure 2).

Most likely due to the presence of submicronic particles (as seen in the TEM image, Figure 2), that could be internalized into the cells, and boost the activation of apoptotic or necrotic processes that resulted in cell viability loss. Xu et al. [20] observed particles with dimensions between 50–300 nm are internalized by endocytosis and also that larger particles (> 500 nm) can be internalized by a phagocytotic mechanism. The incubation of MgAl-HTlc-CO_3_ suspensions with fibroblasts and HepG2 for 24 h did not affect cells viability, in all cases, it remained above 60%. Also, the corresponding solutions were safe for cells testifying to the safety of this HTlc. MgAl-HTlc-Cl resulted also safe for both cell lines as well as the corresponding solutions (Figure 5C,E). It is worth noting that the vitality of HaCat cells was maintained above 60% for all samples. However, the viability of HaCat treated with MgAl-HTlc-Cl and MgAl-HTlc-NO_3_ samples resulted to be more affected than the other two cell lines. Furthermore, when compared to supernatants obtained from increasing HTlc concentrations, the cell viability of MgAl-HTlc-Cl supernatants was reduced (Figure 5B).

This could be ascribed to higher concentrations of either Mg^2+^ and Al^3+^ (in the case of MgAl-HTlc-Cl) ions, measured in DMEM (Figure 3E). Although Mg^2+^ is a necessary component for differentiation, it has been shown that an excess may be harmful to the cell [12]. Furthermore, high doses have been found to have a cytotoxic effect [21,22], triggering apoptosis through mitochondrial impairment [23]. Al^3+^ has been shown to cause morphological changes in keratinocytes as described by Kopa et al. [24].

Thus, it could be hypothesized that HaCat cells are particularly sensitive to the high levels of both metals.

MgAl-HTlc-NO_3_ suspensions resulted in cytotoxic at the two highest concentrations (5.40 and 6.20 mg/mL) for fibroblasts while HepG2 cell viability resulted impaired in the concentration range of 3.80–6.20 mg/mL. The experiment performed by using the supernatant dilutions showed no cytotoxicity after 24 h of incubation for both cell lines suggesting that the cytotoxicity observed for MgAl-HTlc-NO_3_ suspension is attributable to the solid particles and/or to the intercalated nitrate ions as observed by other authors [25,26].

### 2.4. Cytotoxicity Studies on ZnAl-HTlc Samples

**Figure 6:**
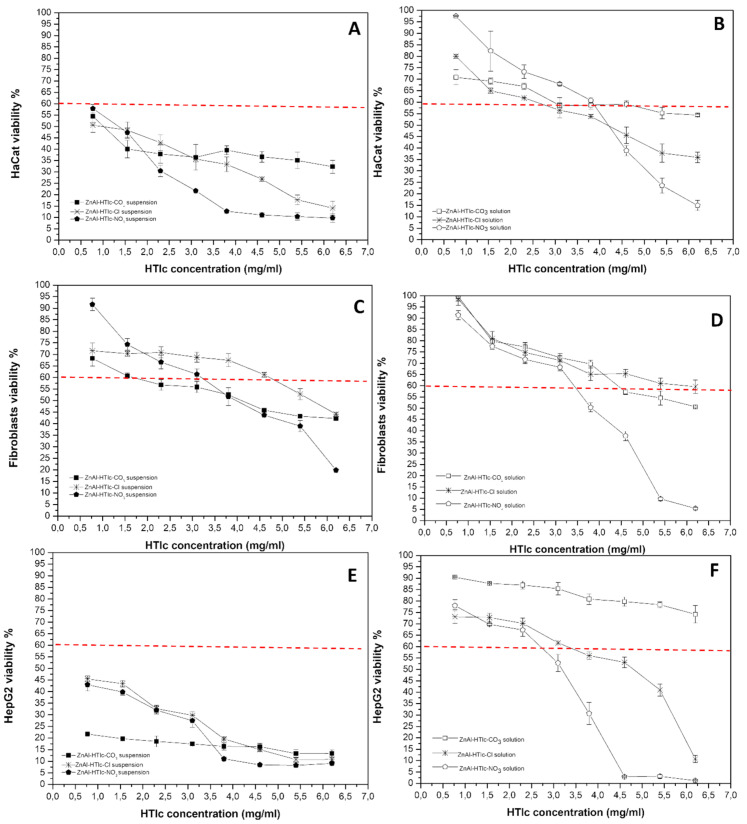
Viability of HaCat cells incubated with ZnAl-HTlc suspensions (**A**) and supernatants (**B**); viability of fibroblasts incubated with ZnAl-HTlc suspensions (**C**) and supernatants (**D**); viability of HepG2 cells incubated with ZnAl-HTlc suspensions (**E**) and supernatants (**F**). The percentage of viable cells with respect to the control was reported as the mean ± SD of five independent experiments. Dotted lines indicate 60% cell viability.

Comparing the cytotoxicity results obtained from ZnAl-HTlc samples (Figure 6) to those obtained from MgAl-HTlc samples (Figure 5), ZnAl-HTlc samples showed a reduction in cell viability, as it already been well described by other authors [11].

For HaCat (Figure 6A) and HepG2 cells, all dilutions of the ZnAl-HTlcs (CO_3_^2−^/Cl^−^/NO_3_^−^) tested suspensions were particularly cytotoxic (Figure 6E). Furthermore, the worst cytotoxicity effect was observed for ZnAl-HTlc-NO_3_ at a concentration range of 3.80–6.20 mg/mL. Similar results were obtained with the HepG2 cell line (Figure 6F). ZnAl-HTlc-CO_3_ and ZnAl-HTlc-Cl) supernatants did not produce cytotoxic effect, except for ZnAl-HTlc-Cl when tested at 3.10 mg/mL. As already observed for the HaCat cell line, ZnAl-HTlc-NO_3_ supernatant induced cell death at the range of 2.30–6.20 mg/mL.

These results suggest that the cytotoxicity observed for the suspensions is attributable to both the solid particles and to the high Zn^2+^ levels measured in the supernatants (Figure 4). As other authors observed, Zn^2+^ are able to induce reactive oxygen species production with consequent apoptosis, once they are penetrated into the cells [27,28].

Different trends were observed for fibroblasts (Figure 6C). ZnAl-HTlc-CO_3_ suspensions resulted in the most cytotoxic, except for the lowest concentration (0.77 mg/mL). ZnAl-HTlc-Cl suspensions were cytotoxic only for the two highest concentrations (5.40 and 6.20 mg/mL) while ZnAl-HTlc-NO_3_ suspensions were cytotoxic at concentration range of 3.80–6.20 mg/mL. By observing the cytotoxicity of the respective HTlc supernatants (Figure 6D), ZnAl-HTlc-Cl did not affect cell viability, whereas ZnAl-HTlc-CO_3_ and ZnAl-HTlc-NO_3_ were cytotoxic at concentrations range of 4.60–6.20 mg/mL and 3.80–6.20 mg/mL, respectively.

As described elsewhere [29], this cell line survives at high Zn^2+^ concentrations owing to interactions of Zn^2+^ with molecules of the extracellular matrix (such as albumin) and so preventing Zn^2+^ from entering inside the cell. In addition, the severe cytotoxicity, observed for the cells treated with the highest ZnAl-HTlc-NO_3_ concentrations, could be attributed to the release of NO_3_^−^ ions.

In the case of HepG2, the suspensions were very cytotoxic while the corresponding supernatants showed different behaviours. In the case of ZnAl-HTlc-CO_3_, Zn^2+^ levels, in MEM (Figure 4A), were lower than ZnAl-HTlc-Cl (Figure 4B) and ZnAl-HTlc-NO_3_ (Figure 4C). Thus, for ZnAl-HTlc-CO_3_ it can be hypothesized that cell death observed for the suspensions is attributable to the solid particles in the medium. As a result, it is plausible that the loss of cell viability occurred with ZnAl-HTlc-CO_3_ suspensions is likely due to the presence of solid particles in the culture media.

In the other cases, the cytotoxicity depended on the presence of high levels of Zn^2+^ in the culture media (Figure 4B,D). Indeed, as in the case of HepG2 [30], high Zn^2+^ levels cause cell death, primarily as a result of mitochondrial damage.

A previous work [31] showed a different behaviour of ZnAl-HTlc nanoparticles on the tumor (HeLa) and normal (MDCK) cells. The authors observed that the nanoparticles are cytotoxic toward the cancer cells as the uptake of this cell is higher compared to the normal one studied. Our findings suggest that by changing the cell line, the behaviour toward a xenobiotic, as nanometric ZnAl-HTlcs are, changed. This supports the idea that the experiments must be performed ad hoc in order to know the safety level of the HTlc material used toward the target tissue.

It is noteworthy that the nanometric forms are able to penetrate the biological membranes such as skin [32]. Especially ZnAl-HTlc could be largely employed in cosmetic products (creams, sunscreens, makeup products), the presence of nanometric forms in the formulation can be harmful to the final user.

This becomes very important especially when the nanometric form is a side product coming from the synthetic procedure and/or from HTlc processing (e.g., milling). In this case, the control of the particle size of the raw HTlc is very important in order to highlight the presence of nanometric fractions in the batch used.

## 3. Materials and Methods

### 3.1. Materials

MgCl_2_ 6∙H_2_O, ZnO, and AlCl_3_ 6∙H_2_O were purchased from Fluka (Milano, Italy). Deionized water was obtained from a reverse osmosis process with Milli Q System (Millipore, Roma, Italy). Other chemicals and solvents were of reagent grade and were used without further purification.

### 3.2. Hydrotalcites Syntheses

Synthetic hydrotalcites MgAl-HTlc-CO_3_ and ZnAl-HTlc-CO_3_ were obtained by adding solid urea to a 0.5 M metal chloride solution (molar ratios: Al^3+^/Al^3+^+M^2+^ = 0.33 and urea/M^2+^+Al^3+^ = 3.3, where M = Mg or Zn) [33]. The hydrolysis of urea, inducing a slow pH increase, led to the precipitation of metals in a well-crystallized HTlc carbonate form. The mixture was heated at 100°C under stirring for 36 h. The final products were recovered by filtration, washed three times with distilled water to eliminate chlorides, dried in an oven at 60°C, and stored in a desiccator over P_2_O_5_ at room temperature. To obtain HTlc with nitrate or chloride anions in the interlayer region (namely: MgAl-HTlc-NO_3_, ZnAl-HTlc-NO_3_, MgAl-HTlc-Cl, ZnAl-HTlc-Cl) the corresponding carbonate forms were first dispersed in a 0.1 M NaCl solution (1 g: 50 mL). The dispersions were then titrated with HNO_3_ 0.1 M or HCl 0.1 M solution by means of an automatic titrator (Radiometer automatic titrimeter, TIM900 Titrilab, and ABU91 Buret); operating in pH-stat mode at pH value of 5 [33].

### 3.3. X-ray Analysis

Powder X-ray diffraction patterns (XRPD) of powders were collected with a Philips X’Pert PRO MPD diffractometer operating at 40 kV and 40 mA, with a step size of 0.03° 2theta, and step scan 40 s, using Cu Kα radiation and an X’Celerator detector. The samples were side-loaded onto a zero-background sample holder to minimize the preferential orientation of microcrystals.

### 3.4. Metal Composition and pH Measurement

HTlcs were incubated for 24 h at 37 °C with the culture media (MEM or DMEM) then the solid particles were separated by centrifugation (4000 rpm for 10 min) and the supernatants were submitted both to pH measurement by a digital pH meter HANNA instruments pH 211and quantification of Mg^2+^, Zn^2+^ and Al^3+^ ions released from HTlcs. Metal analyses were performed by Varian 700-ES series Inductively Coupled Plasma-Optical Emission Spectrometers (ICP-OES) using solutions prepared by dissolving the samples in some drops of concentrated HNO_3_ solution and properly diluted.

### 3.5. Thermogravimetric Analysis (TGA)

Coupled thermogravimetric and differential thermal analyses were performed with a Netzsch STA 449 C apparatus, under a 50 mL min^−1^ in air flow and heating rate of 10 °C/min to determine the weight loss as a function of increasing temperature.

### 3.6. Single Particle Optical Sensing Analysis

The dimensional analysis of HTlc samples was performed using an Accusizer C770 (PSS Inc., Santa Barbara, CA, USA) with the SPOS technique “Single Particle Optical Sensing”. One mg of sample was suspended in 1 mL of bidistilled water, vortexed, and then analysed. The sizes were expressed as cumulative volume distributions (n = 3 ± SD).

### 3.7. SEM and TEM Analyses

The morphology of HTlc particles was studied by Field Emission Scanning Electron Microscopy (FESEM LEO 1525 ZEISS) using an electron high tension of 5 and 15 kV. HTlc samples were prepared by deposition on conductive carbon adhesive tape and then metalized with chromium (8 nm) by sputtering. TEM analysis was performed by a Transmission Electron Microscope (TEM) Philips EM 208. For the analysis, the samples were deposited on a copped grid precoated with Formvar film.

### 3.8. Samples Preparation

HTlcs stock samples were prepared by dispersing 35 mg in 5 mL of cells culture media (DMEM or MEM), obtaining stock solution with a concentration of 7 mg/mL. The obtained suspensions were vortexed, in order to resuspend the solid particles, and then diluted with the culture media in order to obtain different HTlc concentrations (6.20 mg/mL; 5.40 mg/mL; 4.60 mg/mL; 3.80 mg/mL; 3.10 mg/mL; 2.30 mg/mL; 1.55 mg/mL; 0.77 mg/mL). Finally, the obtained dilutions were incubated at 37 °C for 24 h Another experiment was performed in parallel using the supernatant of the stock suspension (7 mg/mL), separated from the latter by centrifugation (10 min at 4000 rpm) diluted with the culture media and the obtained solutions used for the experiments.

### 3.9. Cell Culture

The human immortalized keratinocyte cell line, HaCaT, was purchased from I.Z.S.L.E.R. (Istituto Zooprofilattico Sperimentale della Lombardia e dell’Emilia Romagna) and used as a model to study the epidermal homeostasis and its pathophysiology. In-house established human primary fibroblasts were kindly supplied by Dr. A. Dardis (Regional Coordinator Centre for Rare Diseases, AMC Hospital of Udine, Italy). Both cell lines were cultured according to standard procedures in Dulbecco’s modified Eagle’s medium (DMEM), supplemented with 10% heat-inactivated Fetal Bovine Serum (FBS), 2 mM of L-glutamine, and antibiotics (100 U/mL penicillin, 100 μg/mL streptomycin). HepG2 cells were purchased from ATCC, cultured according to standard procedures in modified Eagle’s medium (MEM), supplemented with 10% heat-inactivated Fetal Bovine Serum (FBS), 2 µM of L-glutamine and antibiotics (100 U/mL penicillin, 100 μg/mL streptomycin). The cells were maintained in a cell incubator at 37 °C in a humidified atmosphere containing 5% CO2. When the cells reached 80–90% of confluence, the routine culture medium was aspirated and the cells were washed two times with PBS 1X. The cells were then harvested by 0.05% trypsin in 0.02% Na_4_EDTA and suspended 1:6 for HaCat, 1:3 for fibroblasts and 1:6 for HepG2 in the proper supplemented growth medium to be maintained in the exponential growth phase. All cells were tested for mycoplasma contamination before use. All studies on human primary fibroblasts were carried out using cells from passages 4–12. For the experiments, the cells were counted by using a trypan blue dye exclusion assay, seeded, and cultured in the absence or presence of samples at different concentrations and for different times. In all experiments, untreated cells were used as negative controls.

### 3.10. Cell Viability

Cellular viability was assessed by the reduction of MTT to formazan [34]. HaCat and human primary fibroblasts were seeded onto a 96-well plate at a density of 3 × 10^3^ cells/well and 1 × 10^4^ cells/well respectively with DMEM complete medium. HepG2 was used in MEM at a density of 1 × 10^4^ cells/well. After 24 h fresh complete medium was replaced for treatment (for 24 h) with different volumes of the supernatants/suspensions obtained from each sample diluted with DMEM to get to the final solution volume of 180 μL. After 24 h the MTT reagent was dissolved in PBS 1X, and added to the culture at 0.5 mg/mL final concentration (20 μL). After 3 h incubation at 37 °C, the supernatant was carefully removed and formazan salt crystals were dissolved in 200 μL DMSO added to each well. After 30 min the absorbance (OD) values were measured spectrophotometrically at 540 nm using an automatic microplate reader (Eliza MAT 2000, DRG Instruments, GmbH). Each experiment was performed two times in triplicate. Cell viability was expressed as a percentage relative to that of the control cells as described previously [35].

## 4. Conclusions

In vitro cytotoxicity studies showed that MgAl-HTlcs and ZnAl-HTlcs, intercalating CO_3_^2−^/Cl^−^/NO_3_^−^ ions affect the viability of keratinocytes, fibroblasts, and hepatocytes. The main factor responsible for cell death is represented by the submicronic fraction, identified in all the samples, that could be dangerous as easily internalisable into cells with consequent vitality decrease. Together with this, in the case of cells treated with ZnAl-HTlcs samples, another factor responsible for the viability impairment is represented by the high concentration of Zn^2+^ deriving from HTlc partial solubilization. The obtained results suggest that the investigated HTlcs cannot be considered absolutely safe and *ad hoc* studies must be performed on the raw material whatever formulation will be developed.

## Figures and Tables

**Table 1:** Interlayer distance measured by XRPD analysis and empirical formula of HTlc.

Sample	Interlayer Distance (Å)	Formula
ZnAl-HTlc-CO_3_	7.5	[Zn_0.65_Al_0.35_(OH)_2_](CO_3_)_0.175_•0.4 H_2_O
ZnAl-HTlc-Cl	7.8	[Zn_0.62_Al_0.38_(OH)_2_](Cl)_0.38_•0.6 H_2_O
ZnAl-HTlc-NO_3_	8.9	[Zn_0.69_Al_0.31_(OH)_2_](NO_3_)_0.31_•0.5 H_2_O
MgAl-HTlc-CO_3_	7.5	[Mg_0.67_Al_0.33_(OH)_2_](CO_3_)_0.165_•0.4 H_2_O
MgAl-HTlc-Cl	7.8	[Mg_0.60_Al_0.40_(OH)_2_](Cl)_0.40_•0.6 H_2_O
MgAl-HTlc-NO_3_	8.9	[Mg_0.63_Al_0.37_(OH)_2_](NO_3_)_0.37_•0.5 H_2_O

**Table 2:** Dimensional values of the different HTlcs by SPOS analysis (*n* = 3; ±SD).

Sample	SPOS			
	D10 (µm) ± SD	D50 (µm) ± SD	D90 (µm) ± SD	Mode (µm) ± SD
MgAl-HTlc-CO_3_	3.99 ± 0.03	8.50 ± 0.11	13.93 ± 0.03	9.47 ± 0.01
MgAl-HTlc-Cl	2.33 ± 0.15	8.50 ± 0.13	16.24 ± 0.17	8.97 ± 0.11
MgAl-HTlc-NO_3_	3.78 ± 0.23	9.47 ± 0.05	35.68 ± 0.19	8.50 ± 0.02
ZnAl-HTlc-CO_3_	8.50 ± 0.13	14.42 ± 0.08	23.70 ± 0.08	13.82 ± 0.05
ZnAl-HTlc-Cl	4.45 ± 0.19	7.63 ± 0.12	13.09 ± 0.09	7.63 ± 0.03
ZnAl-HTlc-NO_3_	3.80 ± 0.22	5.99 ± 0.02	9.01 ± 0.05	5.99 ± 0.06

D10, D50, and D90: size values corresponding to cumulative distributions at 10%, 50%, and 90%, respectively. These represent the particle sizes, below which 10%, 50%, and 90%, respectively, of the samples’ particles lie.

**Table 3:** pH values were measured for the supernatant obtained after incubation of HTlcs with culture media for 24 h at 37 °C (n = 3; ±SD).

Sample	pH (MEM)	pH (DMEM)
MgAl-HTlc-CO_3_	7.50 ±0.01	8.05 ± 0.04
MgAl-HTlc-Cl	7.40 ± 0.02	7.40 ± 0.02
MgAl-HTlc-NO_3_	7.27 ± 0.03	7.45 ± 0.01
ZnAl-HTlc-CO_3_	7.51 ±0.01	8.16 ± 0.03
ZnAl-HTlc-Cl	7.40 ± 0.01	7.45 ± 0.02
ZnAl-HTlc-NO_3_	7.40 ± 0.02	7.46 ± 0.02

## Data Availability

Not applicable.
